# Glycocalyx analysis of bladder cancer: three-dimensional images in electron microscopy and vicia villosa lectin as a marker for invasiveness in frozen sections

**DOI:** 10.3389/fcell.2023.1308879

**Published:** 2024-01-10

**Authors:** Torai Enomoto, Hideshi Okada, Hiroyuki Tomita, Koji Iinuma, Keita Nakane, Yuki Tobisawa, Akira Hara, Takuya Koie

**Affiliations:** ^1^ Department of Urology, Gifu University Graduate School of Medicine, Gifu, Japan; ^2^ Department of Urology, Matsunami General Hospital, Gifu, Japan; ^3^ Department of Emergency and Disaster Medicine, Gifu University Graduate School of Medicine, Gifu, Japan; ^4^ Center for One Medicine Innovative Translational Research, Gifu University Institute for Advanced Study, Gifu, Japan; ^5^ Department of Tumor Pathology, Gifu University Graduate School of Medicine, Gifu, Japan

**Keywords:** glycocalyx, cancer, lectin, urinary bladder cancer, metastasis, scanning electron microscopy, fluorescence microscopy, frozen sections

## Abstract

**Introduction:** The abnormal glycocalyx (GCX) on the surface of cancer cells has been reported to be tall and aberrantly glycosylated and has been linked to the progression and spread of cancer—a finding also observed in bladder cancer. However, the characteristics of GCX in various types of human bladder cancer remain unknown, and herein, we aimed to provide information on the diversity of glycan components in the GCX of bladder cancers and to shed light on their characteristics.

**Methods:** We used scanning electron microscopy and lanthanum staining to examine the surface GCX of human bladder carcinomas in three-dimensional images, showing the bulky GCX in some carcinomas. We also examined glycan alterations in early to progressive stages of bladder cancers using 20 distinct lectin stains on frozen sections from transurethral resection of bladder tumors.

**Results and discussion:** Distinctive Vicia villosa lectin (VVL) staining was observed in invasive urothelial carcinomas, including those with muscle invasion and variant components. In the clinical setting, cancers with atypia of grades 2–3 had a significantly higher VVL scoring intensity than those with grade 1 atypia (*p* < 0.005). This study identified that a specific lectin, VVL, was more specific to invasive urothelial carcinomas. This lectin, which selectively binds to sites of cancer progression, is a promising target for drug delivery in future clinical investigations.

## Introduction

Bladder cancer is the 10th most common cancer worldwide (Sung et al., 2021). In Japan, in 2019, approximately 23,000 individuals were newly diagnosed with bladder cancer and approximately 9,000 people died because of it (National Cancer Center, 2020). Men are twice as likely to develop bladder cancer as women; furthermore, bladder cancer is associated with various risk factors, such as geography, age, and exposure to carcinogens, and smoking is the most influential risk factor (Cumberbatch et al., 2016). The 5-year relative survival rates for stage I, stage II, stage III, and stage IV bladder cancer are 86.4%, 57.0%, 43.1%, and 19.3%, respectively, with significantly lower survival rates for muscle-invasive and/or metastatic bladder cancers (National Cancer Center, 2020). Approximately 75% of bladder cancers are non-invasive or stromal invasive at diagnosis, and approximately 25% are muscle-invasive bladder cancers (Burger et al., 2013; Catto et al., 2021; Witjes et al., 2021). The rate of progression of non-muscle invasive bladder cancer to muscle layer invasion has been reported to be 15%–50% (Sylvester et al., 2006). Administering neoadjuvant chemotherapy before a radical cystectomy for muscle-invasive bladder cancer with variant histology improves patient survival (Vetterlein et al., 2017). Therefore, the presence or absence of muscle invasion is a discriminating factor in determining the prognosis and treatment plan (Burger et al., 2013).

Most bladder cancers are characterized by the presence of transitional epithelial cells. Bladder cancer can be of various types: squamous cell, adenoblastic cell, undifferentiated cell, focal cell, micropapillary cell, lymphoepithelial tumor, plasmacytoid, and sarcomatoid (Chalasani et al., 2009). Histologic variants of urothelial carcinoma comprise tumors arising from within the urothelium, in which some components of the tumor morphology are other than the urothelium (Chalasani et al., 2009). The micropapillary, plasmacytoid, nested, and sarcomatoid histologic variants of urothelial carcinoma have been linked with poorer survival outcomes than conventional urothelial carcinomas and urothelial carcinomas with squamous or glandular differentiation, despite undergoing chemotherapy-induced downstaging (Ogbue et al., 2022).

The cell surface is typically covered with a glycocalyx (GCX) structure, which is a protective and interactive molecular coating on cell surfaces, composed of proteoglycans, glycosaminoglycans, mucins, glycoproteins, and glycolipids, essential for cell communication, stability, and defense (Buffone and Weaver, 2020). The function and structure of GCX exhibit considerable variability among different cellular, tissue, and organ types, with notable diversity influenced by specific environmental conditions (Salmon and Satchell, 2012). The GCX on the surface of cancer cells is reported to be tall and aberrantly glycosylated (Tarbell and Cancel, 2016; Tachi et al., 2019), and this alteration in GCX has been linked to the progression and metastasis of cancer through multiple mechanisms (Hammond et al., 2014). Another report suggests that cancer cells exhibit an augmented GCX density, leading to heightened cell membrane tension, modified tissue mechanics, and the promotion of a more malignant phenotype (Buffone and Weaver, 2020). When the cancer glycocalyx is described as bulky, it typically suggests that there is an increased or excessive accumulation of carbohydrates, particularly in the form of glycoproteins, in this outer layer (Hakomori, 1996; Paszek et al., 2014). Many types of cancer cells overexpress bulky glycoproteins to form a thick glycocalyx layer.

GCX consists of several carbohydrate moieties of proteoglycans, glycosaminoglycans, mucins, glycoproteins, and glycolipids, and the specific carbohydrate-binding activity of lectins is used to identify the location of the carbohydrate chains (Suzuki et al., 2022). Lectin staining is used not only to assess the thickness of GCX on the cell surface, but also the type and amount of constituent glycans of that GCX. A panel of biotin-labelled plant lectins has been used to detect a broad spectrum of glycan motifs with high specificity. There have been many reports evaluating glycan changes in bladder cancer progression using lectins; however, the outcomes have been inconsistent (Sumiya et al., 1988; Yagi, 1989; Huo et al., 2022). This may be attributed to the method of tissue fixation, as glycans are unstable and formalin-fixed paraffin-embedded (FFPE) specimens have been reported to be less responsive to lectins than frozen specimens (Brandon et al., 1988).

This study aimed to provide information on the diversity of glycan components in the GCX of bladder cancers and to shed light on their characteristics; it also aimed to visualize cell surfaces of bladder cancer covered with bulky GCX with three dimensional (3D) images using scanning electron microscopy (SEM). We hypothesized that this may clarify the clinical significance of GCX in urinary bladder cancers.

## Materials and methods

### Patients

Fresh surgical specimens of seven patients were included in the study. Five patients had primary bladder cancers, two had lymph node metastasis, and one had peritoneal metastasis. The following histopathological diagnoses were assigned to the seven patients: non-invasive urothelial carcinomas (*n* = 2), invasive urothelial carcinomas (*n* = 2), muscle invasive urothelial carcinomas (*n* = 2; sarcomatoid variant [*n* = 1], and plasmacytoid variant [*n* = 1]), and small-cell carcinoma (*n* = 1). The mean patient age was 75 ± 6 years (range, 67–84 years). The tumor stages were as follows: stage 0a (*n* = 2), stage I (*n* = 3), and stage IV (*n* = 2).

The study also included 59 FFPE tissue sections from 57 patients. Fifty-three patients had primary bladder cancer, and four had metastatic bladder cancer. Twenty patients underwent repeat TURBT due to recurrence. Of the 59 sections, 31 were pathologically diagnosed as “non-invasive urothelial carcinomas,” 17 as “invasive urothelial carcinomas,” 10 as “muscle invasive urothelial carcinomas,” and one as “small-cell carcinoma.” The twenty of the 27 invasive and muscle-invasive urothelial carcinomas were conventional and the remaining seven were of the following variants: glandular variant (*n* = 1), micropapillary variant (*n* = 1), squamous differentiation (*n* = 3), and sarcomatoid variant (*n* = 3). One patient had a combination of squamous differentiation and a sarcomatoid variant. The mean age of the 57 patients was 73 ± 10 years (range, 46–87). In the 59 sections, the following stages were observed: stage 0a (*n* = 30), stage 0is (*n* = 1), stage I (*n* = 18), stage II (*n* = 5), stage III (*n* = 2), and stage IV (*n* = 3).

All patients underwent TURBT at the Department of Urology, Gifu University Hospital, Japan between 2020 and 2021. None of the patients had received chemotherapy before surgery. Tumors were staged according to the criteria of the General Rule for Clinical and Pathological Studies on Renal, Pelvic, Ureteral, and Bladder Cancer, second Edition. Informed patient consent and prior approval from the Gifu University Hospital Affiliated to Gifu University Graduate School of Medicine Ethics Committees (approval no. 2020–256) were obtained before the clinical materials were used for research purposes.

### Frozen and FFPE sections of tissue specimens

For the preparation of frozen sections, surgical specimens were collected within 60 min of surgery. Each sample consisted of approximately 4-mm cubes of tissue. Tissues were protected with Tissue OCT compound (Sakura Finetek, Japan), snap frozen in liquid nitrogen, and stored at −80°C. Next, 5-μm serial sections were cut using a Leica CM1850 cryostat (Leica Microsystems, Wetzlar, Germany). Sections were air-dried at room temperature for 30 min; one slide was stained with hematoxylin and eosin (HE), and other slides were stored at −20°C until use.

For the preparation of FFPE sections, surgical specimens were fixed in 10% neutral buffered formalin, routinely processed, and whole-mount embedded. The HE-stained sections were histologically evaluated at the Department of Pathology, Gifu University Hospital, Japan.

### Scanning electron microscopy (SEM)

The tissue samples were diced into approximately 1-mm^3^ sized cubes. Then, some of these tissue pieces were light-shielded and soaked overnight in 2% glutaraldehyde at 4°C. To observe GCX on the surface of bladder cancer cells, the rest of these tissue pieces were immersed in a solution composed of 2% glutaraldehyde, 2% sucrose, 0.1 M sodium cacodylate buffer (pH 7.3), and 2% lanthanum nitrate for 2 h; later, they were soaked overnight in a solution without glutaraldehyde and washed in alkaline (0.03 M NaOH) sucrose (2%) solution. The specimens were then dehydrated using a graded ethanol series. The frozen fracture method was used to prepare the samples for 3D examination using SEM. Each sample was placed on an iron plate chilled with liquid nitrogen, and ethanol was sprinkled onto it. Once the ethanol was frozen, the sample was fractured using a chisel, such that it was not directly touched. The samples were incubated with tert-butyl alcohol at room temperature. After the tert-butyl alcohol solidified, it was freeze-dried, and the specimens were examined using SEM (S-4800; Hitachi, Tokyo, Japan).

### Lectin staining with immunofluorescent staining for frozen samples

Twenty different types of lectins (lectin screening kits; I-III, Vector Laboratories, Newark, California, United States) used in the present study are summarized in Table 1. These lectin screening kits are comprehensive kits for the detection of glycan expression in tissue sections. Lectins were classified into five groups according to the binding specificity and inhibitory sugars, including N-acetylglucosamine, mannose, galactose/N-acetylgalactosamine, complex type N-glycan groups (PHA-E and PHA-L), and fucose as discussed and well-considered in previous reports (Kaltner et al., 2007; Ibrahim et al., 2013; Kang et al., 2016).

**TABLE 1:** The 20 lectins used in this study and the corresponding glycans specifically recognized.

By the lectins	Common abbreviation	Primary sugar specificity
Concanavalin A	Con A	Mannose
Dolichos biflorus agglutinin	DBA	N-Acetylgalactosamine
Peanut Agglutinin	PNA	Galactose
Ricinus communis agglutininI	RCAI	Galactose, N-Acetylgalactosamine
Soybean agglutinin	SBA	N-Acetylgalactosamine
Ulex Europaeus agglutininI	UEAI	Fucose
Wheat Germ agglutinin	WGA	N-Acetylglucosamine
Griffonia simplicifolia lectinI	GSLI	Galactose
Len culinaris lectin	LCA	Mannose
Phaseolus vulgaris Erythroagglutinin	PHA-E	Complex structures
Phaseolus vulgaris Leucoagglutinin	PHA-L	Complex structures
Pisum sativum agglutinin	PSA	Mannose
Wheat Germ agglutinin, Succinylated	Succinylated WGA	N-Acetylglucosamine
Datura stramonium lectin	DSL	N-Acetylglucosamine
Erythrina cristagalli lectin	ECL	Galactose
Griffonia simplicifolia lectinII	GSLII	N-Acetylglucosamine
Jacalin	Jacalin	Galactose
Lycopersicon esculentum lectin	LEL	N-Acetylglucosamine
Solanum tuberosum lectin	STL	N-Acetylglucosamine
Vicia villosa lectin	VVL	N-Acetylgalactosamine

For frozen section staining, fresh tissue sections were fixed for 15 min in 4% paraformaldehyde (0.1 M phosphate buffered saline [PBS], pH 7.4). Then they were then soaked in phosphate buffered saline for 10 min, placed in Carbon Free Blocking Solution (CFBS; Vector Laboratories), and maintained at room temperature for 60 min. Next, the mixture of biotinylated-lectin and a rabbit monoclonal anti-CD 31, an endothelial cell marker, antibody (ab76533; Abcam, Cambridge, United Kingdom), which was diluted 200 times with PBS, was added, and kept overnight in a refrigerator at 4°C. The next day, after immersion of the sections in PBS for 15 min, streptavidin-Dylight 594 (Vector Laboratories) and goat anti-Rabbit IgG H&L-Dylight 488 (ab96899; Abcam), which was diluted 200 times with PBS, was added, and maintained at room temperature for 60 min. After immersion in PBS for 15 min again, sections were immersed in 4′, 6-Diamidino-2-phenylindole, dihydrochloride (DAPI; DOJIDO) for 5 min. The sections were then immersed in PBS for 5 min, sealed with a fluorescent anti-extinguishing sealant, and observed under a microscope (BX53; OLYMPUS, Japan).

### Vicia villosa lectin (VVL) staining for FFPE samples

For paraffin section staining, tissue sections were incubated at 65°C for 40 min and maintained at room temperature for 5 min. For deparaffinization, the sections were immersed in xylene for 7 min, washed with alcohol, and washed with water for 2 min. They were then immersed in 0.01 M citric acid buffer, and after 30 min of the antigen retrieval by heating, they were cooled at room temperature for 1 h. Next, sections were immersed in PBS for 15 min, soaked in 3% methanol for 10 min, and again soaked in PBS for 15 min for permeabilization. Sections were placed in CFBS and kept at room temperature for 40 min. They were then added the biotinylated-VVL that was diluted 200 times and kept overnight in a refrigerator at 4°C. The slides were washed with PBS, added the ABC reagent (PK-7100, Vector Laboratories, United States) and maintained at room temperature for 60 min. The slides were washed with PBS and added with DAB (Sigma-Aldrich, St. Louis, Missouri, United States). They were counterstained by hematoxylin, sealed with a cover slip, and observed under a microscope.

### Assessment of immunostaining results

Immunostaining results were assessed by staining intensity (0–3) for the tested tissues using a microscope using the 4× to 40× objective lenses. The criteria for assessment were defined as follows: negative (0); weak (1); moderate (2); and strong (3). The vast majority of cases exhibited a uniform degree of staining among all tested samples, and an average score was determined for those that did not. Evaluation of tissue staining was performed independently by two experienced observers. All specimens were scored blindly, and an average of the scored was measured. For statistical analysis, all cases were divided into 2 groups: the negative group, composed of staining intensity 0, and the positive group, composed of staining intensity 1, 2 and 3.

### Quantitative scoring of lectin-staining intensity

For quantitative analysis of fluorescent intensity, scoring was performed using ImageJ software. The intensity score was scored manually in 40 high-power fields per sample (*n* = 3 per sample), in the focal plane as previously described (Suzuki et al., 2020).

### RNA extraction and real time RT-PCR

OCT-embedded frozen tissues were cut into 20 μm sections. Total RNA was isolated from the 10 sections per one sample using the RNeasy Mini Kit (QIAGEN GmbH, Hilden, Germany) according to the manufacturer’s protocol. A Superscript III kit (Life Technologies, United States) was used for reverse transcription (RT), and the resulting cDNA was used for qRT-PCR. The cDNA was subjected to real-time PCR analysis of gene expression using SYBR Green Premix Ex Taq (TaKaRa, JAPAN) on a StepOne Real-time PCR machine (Applied Biosystems, CA, United States). Each group was subjected to a minimum of two distinct experiments. 2^−ΔΔCT^ (ΔCT = CT_target gene_–CT_housekeeping gene_) was used to determine the relative gene expression level. The used primers are listed in Supplementary Table S1.

### Statistical analysis

Associations between VVL staining intensity and clinicopathological data were tested using the Chi-squared test. All *p* values were 2-sided and *p* < 0.05 was considered statistically significant. All statistical analyses were performed using GraphPad Prism version 9.5.1.

## Results

### Bulky GCX of human bladder cancers was visualized in the 3D SEM images

**FIGURE 1:**
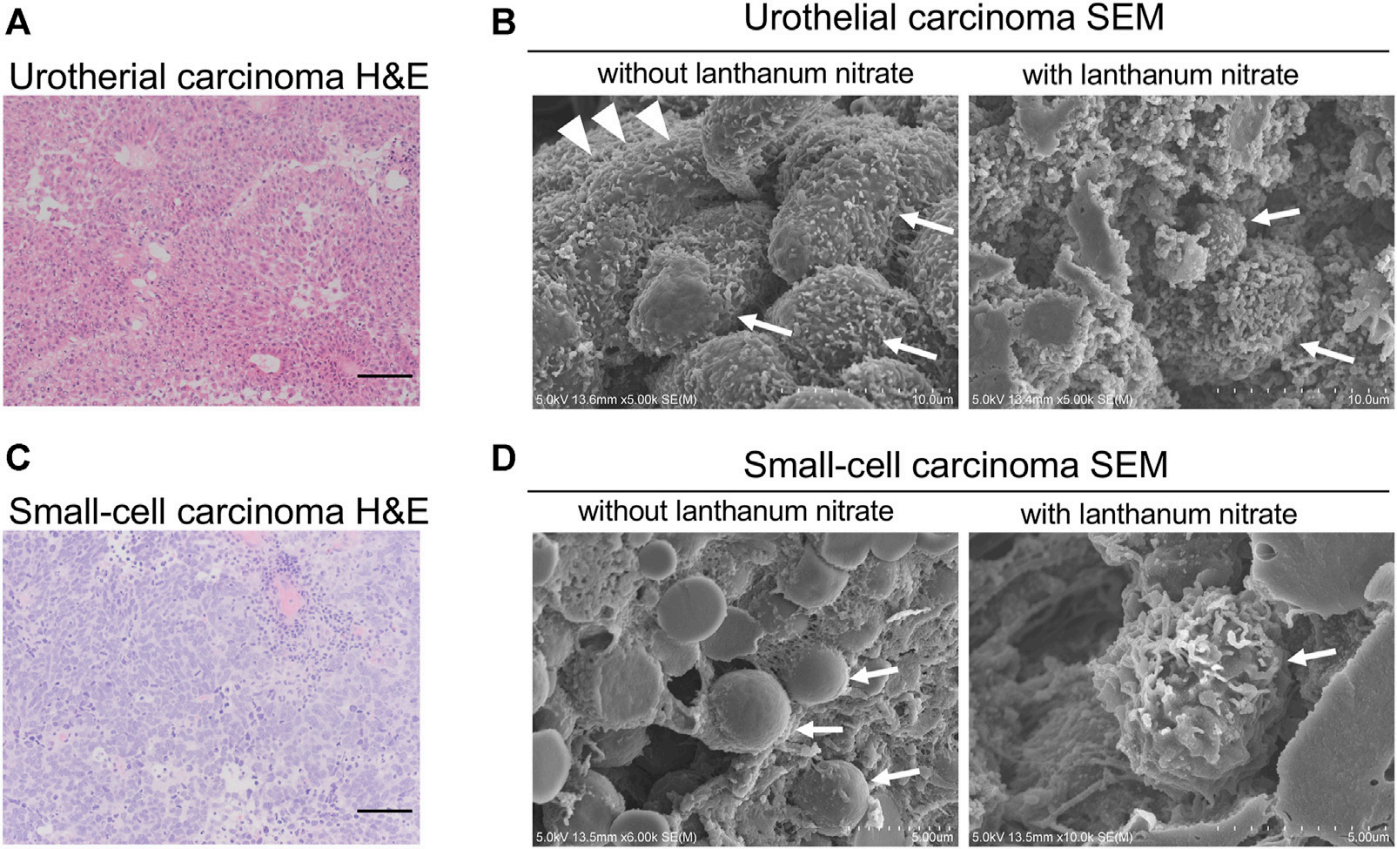
SEM images of the cancer cell surfaces with and without lanthanum staining **(A)** Urothelial carcinoma, HE staining; **(B)** Urothelial carcinoma examined using SEM; **(C)** Small-cell carcinoma, HE staining; **(D)** Small-cell carcinoma examined using SEM. “With lanthanum nitrate” indicates the GCX depiction. “Without lanthanum nitrate” indicates that the GCX is not captured. White arrows indicate individual cancer cells. White arrowheads indicate microvilli. Scale bars = 100 μm.

To visualize the ultrastructure of GCX on the surface of human bladder cancer cells, fresh human tissues were fixed and observed by SEM. We observed the GCX structures in two different bladder cancers: non-invasive urothelial carcinoma (Figure 1A) and small-cell carcinoma (Figure 1C).

Lanthanum staining allows visualization of the 3D features of GCX, while the cell surface without lanthanum staining allows observation of the cancer cell surface without GCX. In non-invasive urothelial carcinoma without GCX, abundant microvilli were observed on the cell surface (Figure 1B). On the other hand, the surface of small cell carcinoma witout GCX was smooth without microvilli (Figure 1D). Both non-invasive urothelial carcinoma and small cell carcinoma were shown to have bulky and abundant GCX on the cancer cell surface (Figures 1B, D).

The 3D ultrastructures of GCX indicated moss-like growth on the cancer cell surface. These results suggest that human bladder cancers have bulky GCX on the cell surface.

### Among the 20 lectins, VVL staining was distinctly observed in invasive urothelial carcinomas

In this study, we focused on the morphological and microscopic characteristics of the cancer surface glycocalyx, which is bulky, such as taller and longer than normal cells. Thus, histopathological analysis using tissue specimens is required. To investigate the glycan components of the bulky GCX in bladder cancers, fresh tissues were obtained and stained with 20 different lectins (Table 1). Fresh tissues from human bladder tumors were obtained from seven cases of bladder cancer (five localized cancers and two metastatic cancers) obtained via TURBT (Table 2).

**TABLE 2:** Patient data for frozen and FFPE tissue sections of bladder cancer.

	Frozen section	Paraffin section
Number of patients (sections)	7 (7)	57 (59)
Age mean ± SD (years)	75 ± 6 years	73 ± 10 years
Age range	67–84	46–87
Sex		
male	5 (71%)	45 (79%)
female	2 (29%)	12 (21%)
Clinical stage		
0a	2 (29%)	30 (51%)
0is	0	1 (2%)
I	3 (42%)	18 (31%)
II	0	5 (8%)
III	0	2 (3%)
IV	2 (29%)	3 (5%)
Tumor classification		
a	2 (29%)	30 (51%)
Cis	0	1 (2%)
1	3 (42%)	18 (31%)
2	0	5 (8%)
3	1 (14%)	4 (6%)
4	1 (14%)	1 (2%)
Node classification		
0	5 (71%)	56 (95%)
1	2 (29%)	2 (3%)
2	0	0
3	0	1 (2%)
metastasis classfication		
0	6 (86%)	56 (95%)
1	1 (14%)	3 (5%)
Grading		
1	2 (29%)	13 (22%)
2	3 (42%)	32 (54%)
3	2 (29%)	14 (24%)
Histology		
Urothelial carcinoma	6 (86%)	58 (98%)
small cell carcinoma	1 (14%)	1 (2%)
Invasiveness		
non-invasive	2 (29%)	31 (53%)
invasive	5 (71%)	28 (47%)
Variant/differentiation		
glandular differentiation	0	1 (4%)
squamous differentiation	0	3 (11%)
sarcomatoid variant	1 (14%)	3 (11%)
micropapillary variant	0	1 (4%)
plasmacytoid variant	1 (14%)	0

**FIGURE 2:**
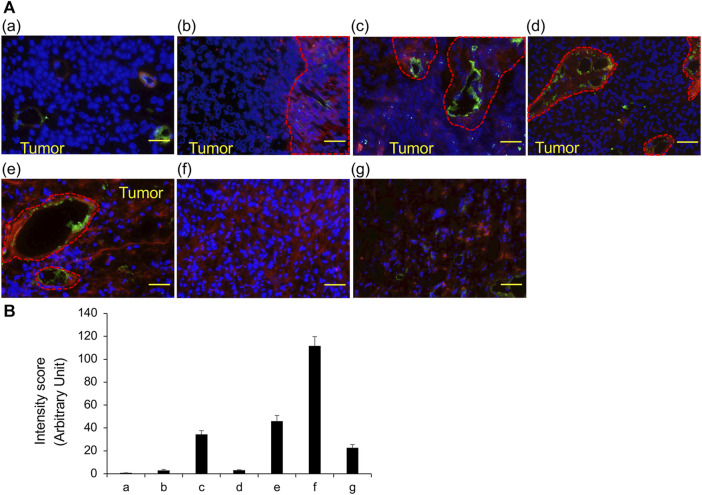
VVL staining of frozen sections obtained from TURBT observed using fluorescence microscopy **(A)** The representative fluorescent images of bladder cancers. VVL (*Red*), CD31 (*Green*) and nuclei (*Blue*) are shown. **(a)** Low-grade non-invasive urothelial carcinoma; **(b)** Low- and high-grade non-invasive urothelial carcinoma; **(c)** High-grade invasive urothelial carcinoma; **(d)** High-grade invasive urothelial carcinoma; **(e)** Muscle invasive urothelial carcinoma, sarcomatoid variant; **(f)** Muscle invasive urothelial carcinoma, plasmacytoid variant; **(g)** Small-cell carcinoma. Scale bars = 50 μm. **(B)** Scoring of VVL staining intensity was performed using the ImageJ software. Data represent the mean ± SEM of each three spot of each photo.

The results of staining frozen sections with 20 different lectins are summarized in Table 3; ConA, LCA, and PSA predominantly bind to mannose, while WGA, DSL, GSL II, LEL, and STL predominantly bind to N-acetylglucosamine; these lectins were identified in almost all cancer cells of all tissue sections. In contrast, DBA, RCA I, and SBA predominantly bind to N-acetylgalactosamine; ECL and Jacalin predominantly bind to galactose; and succinylated WGA predominantly binds to N-acetylglucosamine; these lectins were not identified in any of the tissue sections. PNA and GSL I, which bind to galactose predominantly, were not observed in invasive urothelial carcinoma but were seen in sections of urothelial carcinoma with variant components. We confirmed the results of VVL by measuring the fluorescent intensity (Figure 2). These results indicate that VVL may serve as a specific glycan marker for the identification of invasive urothelial carcinomas.

**TABLE 3:** Staining profiles of the 20 lectins for frozen sections obtained from TURBT.

	Non-invasive urothelial carcinoma		Invasive urothelial carcinoma		Muscle invasive urothelial carcinoma		Small cell carcinoma
	Low grade	Low and high grade	High grade	High grade	Sarcomatoid variant	Plasmacytoid variant	
ConA	3	3	3	3	3	3	3
DBA	1	2	0	0	1	0	2
PNA	0	1	0	0	0	1	3
RCAI	0	3	3	2	3	3	0
SBA	0	0	0	0	1	0	0
UEAI	0	1	0	2	0	0	3
WGA	3	3	2	3	3	3	0
GSLI	2	2	1	0	2	2	2
LCA	3	3	2	1	0	1	2
PHA-E	3	3	2	3	3	3	3
PHA-L	2	3	0	2	3	3	2
PSA	3	3	3	1	2	2	0
Succinylated WGA	0	0	0	0	0	1	0
DSL	3	3	1	2	3	3	3
ECL	2	3	1	3	3	3	3
GSLII	2	3	0	2	0	2	1
Jacalin	3	2	3	3	3	3	2
LEL	1	3	1	2	3	3	3
STL	1	1	2	2	3	3	2
VVL	0	0	1	0	2	3	1

### VVL staining was generally consistent with frozen and FFPE sections

**FIGURE 3:**
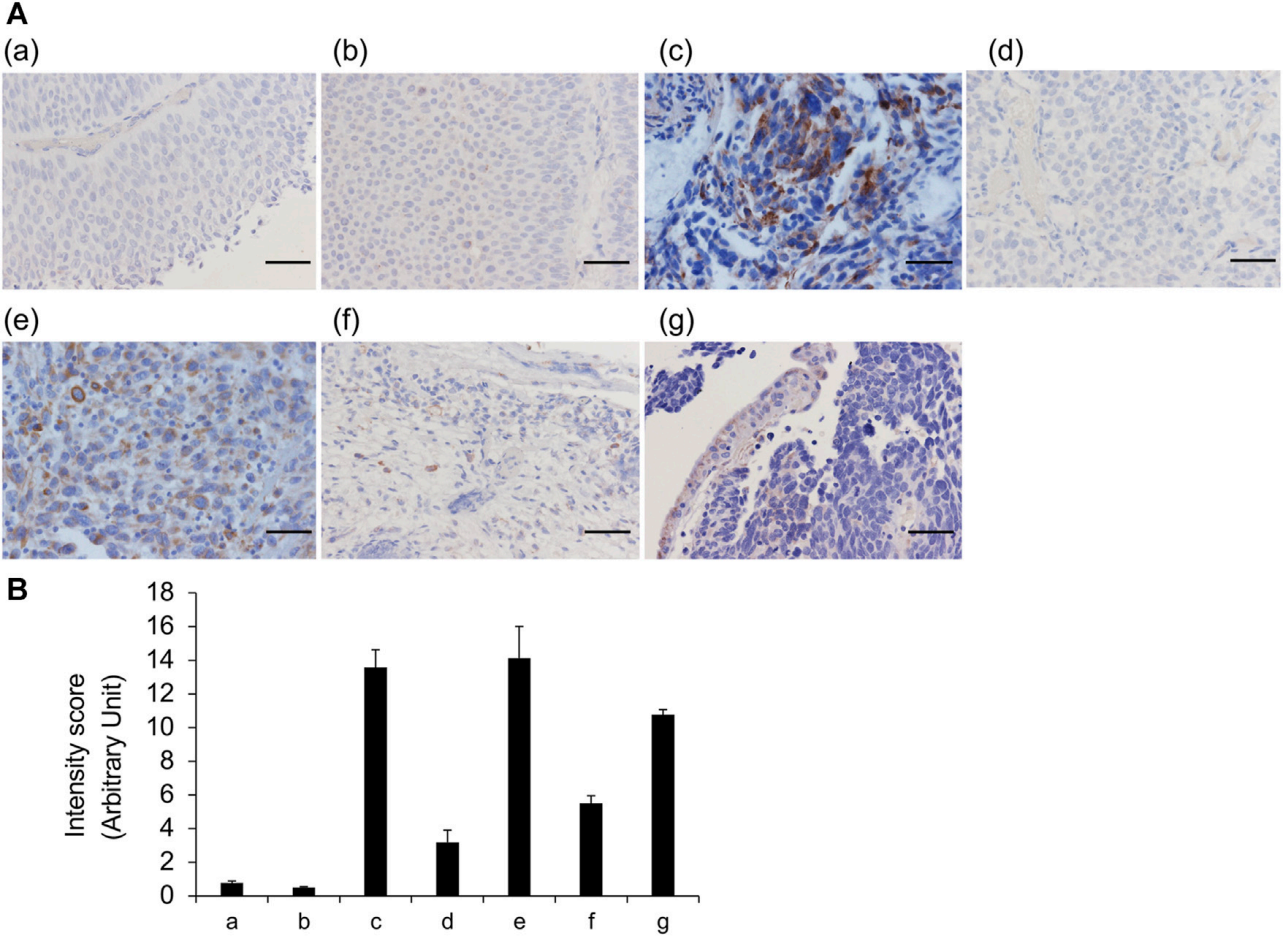
VVL staining of frozen and FFPE sections **(A)** Representative images of bladder cancers. VVL (*brown*) and nuclei (*blue*) are shown. Areas circled by red dotted lines indicate blood vessels. **(a)** Low-grade non-invasive urothelial carcinoma; **(b)** Low- and high-grade non-invasive urothelial carcinoma; **(c)** High-grade invasive urothelial carcinoma; **(d)** High-grade invasive urothelial carcinoma; **(e)** Muscle invasive urothelial carcinoma, sarcomatoid variant; **(f)** Muscle invasive urothelial carcinoma, plasmacytoid variant; **(g)** Small-cell carcinoma. Scale bars = 50 μm. **(B)** Scoring of VVL staining intensity was performed using the ImageJ software. Data represent the mean ± SEM of each three spot of each photo.

Although FFPE tissue sections have tissue definitive morphology upon the microscopic observation, they likely to incur a loss of glycans owing to tissue shrinkage during fixation and treatment. Furthermore, although obtaining a sufficient number of specimens is difficult, frozen tissue sections do not have artifacts. Thus, we analyzed the differences in the staining features of VVL between frozen and FFPE sections for the same seven cases; no noteworthy differences were observed in the staining results between the two types of sections (Figure 3). These data indicated that VVL staining is useful for evaluating VVL in FFPE tissues and frozen sections.

### VVL was highly expressed in invasive urothelial carcinomas with aggressive features

**FIGURE 4:**
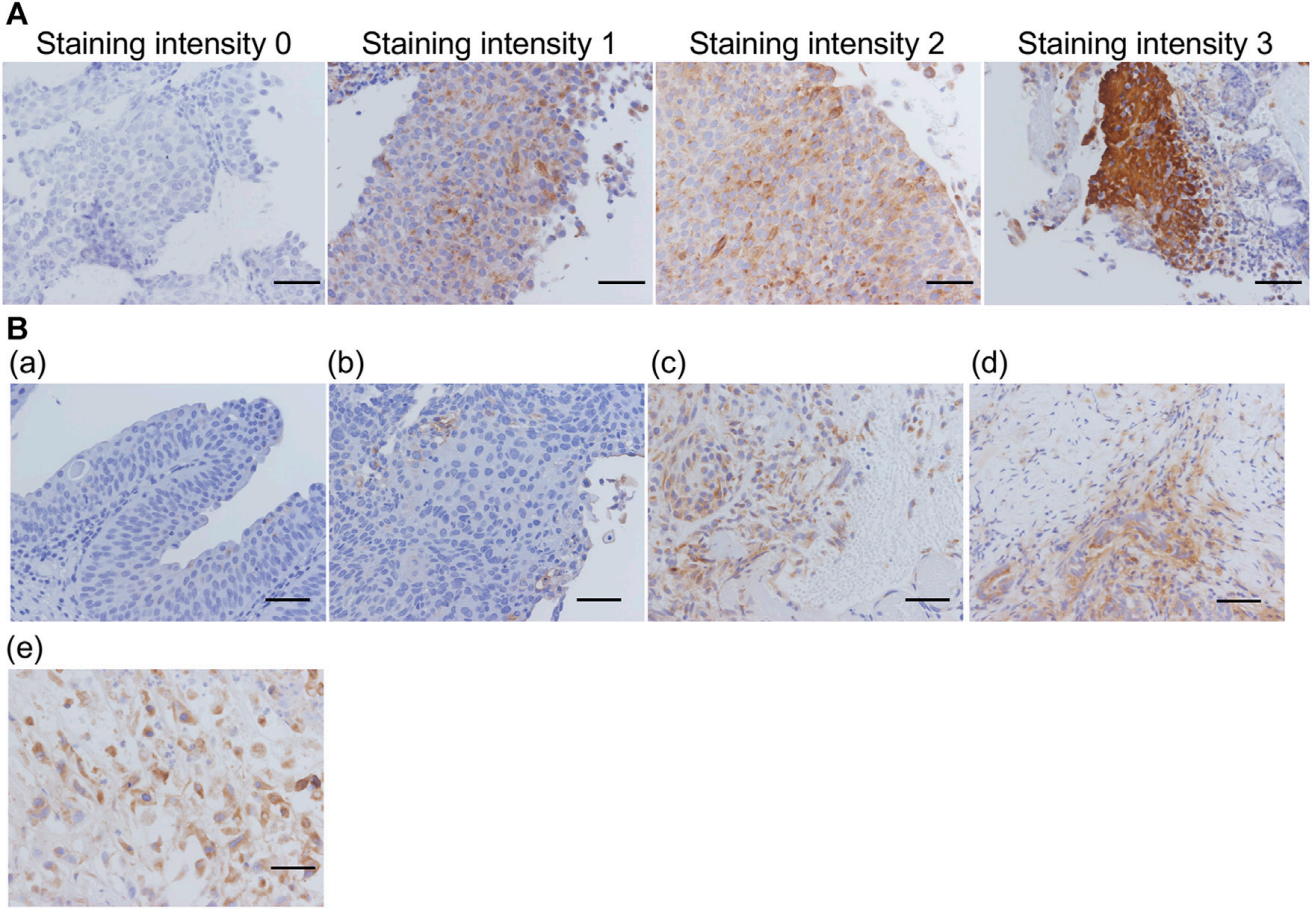
Representative images for each VVL staining score and degree of invasion **(A)** Representative images of VVL staining intensity 0–3. **(B) (a)** Low-grade non-invasive urothelial carcinoma; **(b)** High-grade non-invasive urothelial carcinoma; **(c)** Invasive urothelial carcinoma; **(d)** Muscle invasive urothelial carcinoma; **(e)** Muscle invasive urothelial carcinoma, sarcomatoid variant. Scale bars = 50 μm.

As aforementioned, 59 FFPE sections were prepared from 57 patients, stained with VVL lectin, and their staining intensity were evaluated (Figure 4). Thirty-two sections (54%) were unstained (staining intensity 0); 11 (19%) were weakly stained (staining intensity 1); 4 (6%) were moderately stained (staining intensity 2); and 12 (21%) were strongly stained (staining intensity 3). Some non-invasive urothelial carcinomas had a staining intensity 1, but staining intensity 2 and 3 were more common in invasive urothelial carcinomas, muscle invasive urothelial carcinomas, and urothelial carcinomas with variant components.

Consequently, more intense VVL staining was observed with invasive or muscle invasive urothelial carcinomas and urothelial carcinomas with variant components than that in non-invasive urothelial carcinomas (Figure 4B). With reference to the clinical data (Table 4), there was a significant difference (*p* < 0.05) in VVL staining scores between non-invasive urothelial carcinomas and other type of bladder cancers. A significant difference in VVL staining scores was found between cases with atypia grades 2–3 compared to those with atypia grade 1 (*p* < 0.005). There was no significant difference in VVL staining scores in tumors of the early (0-I) or late (II-IV) stages, those with or without variant components in pathology, and those with or without metastasis.

**TABLE 4:** Clinicopathological profile for FFPE sections obtained from TURBT and stained with VVL.

	Staining intensity		*p*-value
	Negative (0)	Positive (1∼3)	
Non-invasive	21	10	0.0285*
Invasive	11	17	
Clinical stage			
Early (0∼1)	28	21	0.3214
Late(II∼IV)	4	6	
Histology			
Urothelial carcinoma	29	22	0.3068
Others or Variant	3	5	
Metastasis classfication			
0	31	24	0.2241
1	1	3	
Grading			
1	29	16	0.0048**
2–3	3	11	

*p < 0.05, **p < 0.01.

### Upregulation of GALNT 3, 4, 5, 6, and 11 was associated with the VVL-related invasiveness

**FIGURE 5:**
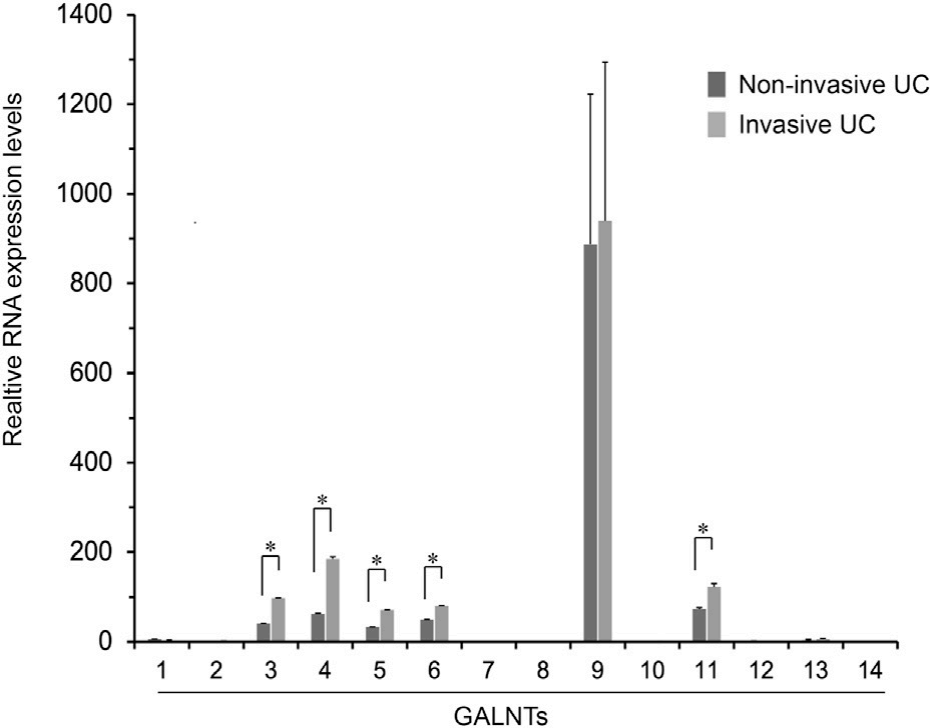
Fold changes in the expression of GALNT1 through GALNT14 in the non-invasive urothelial carcinoma (UC) relative to the invasive UC tissues. Expression levels of GALNT isoforms in the non-invasive and invasive UCs (*n* = 2 each) were determined using real-time RT-PCR of duplicate and two independent experiments. Results are mean ± SEM. *p < 0.05 by Mann-Whitney *U* test.

O-Linked α-N-acetylgalactosamine (O-GalNAc) glycans constitute a major part of the human glycome and VVL is a lectin that recognizes O-GalNAcylated glycans (Detarya et al., 2020). O-GalNAcylation is one class of O-glycosylation that is initiated by the transfer of GalNAc from UDP-GalNAc to acceptor proteins by N-acetyl galactosaminyl transferases (GALNTs) (Ten Hagen et al., 2003; Schjoldager and Clausen, 2012). Thus, to identify GALNTs that contribute to the urothelial carcinoma invasion, we measured GALNT1 to 14 isoforms expressions were examined using real-time RT-PCR. These results showed that RNA expressions of GALNT3, 4, 5, 6, and 11 were upregulated in the invasive compared with non-invasive UC tissues (*p* < 0.05) (Figure 5), suggesting that, at least, several GALNTs expression may be associated with the VVL-related invasiveness in UCs.

## Discussion

In this study, we visualized changes in GCX in bladder cancer cells using 3D SEM and examined glycan expression in frozen and FFPE sections. We found that VVL staining was specific to cases of invasive urothelial carcinoma and its variant components.

The cell surface is typically covered with a glycocalyx (GCX) structure, which is a protective and interactive molecular coating on cell surfaces, composed of proteoglycans, glycosaminoglycans, mucins, glycoproteins, and glycolipids, essential for cell communication, stability, and defense (Buffone and Weaver, 2020). On the surface of cancer cells, GCX is enriched with hyaluronic acid and heparan sulfate and is reported to be bulky (Tarbell and Cancel, 2016); this change in GCX is attributed to contribute to cancer progression and metastasis. For example, heparan sulfate on the cancer cell surface acts as a mechanosensor for interstitial flow that upregulates matrix metalloproteinases release to degrade the extracellular matrix and enhance metastasis (Qazi et al., 2013). Heparan sulfate has also been shown to be required for tumor angiogenesis (Fuster et al., 2007).

Few studies have used electron microscopy to observe changes in GCX in cancer cells (Ramaker et al., 2016). Previous studies have used transmission electron microscopy with lanthanum-based electron staining to describe irregular GCX covering the cell surface in oral squamous cell carcinoma (Palekar and Sirsat, 1975); they demonstrated that GCX on the cancer cell surface is different in each type of tumor cell, using stimulated emission depletion microscopy (von Palubitzki et al., 2020). Herein, we were able to successfully visualize GCX on the surface of bladder cancer cells in three dimensions using SEM. Despite the differences in cell surface morphology between urothelial and small-cell carcinomas, both carcinomas had bulky GCX. The term, bulky GCX, in cancer cells typically suggests that there is an increased or excessive accumulation of carbohydrates, particularly in the form of glycoproteins, in the outer layer of cancer cells (Hakomori, 1996; Paszek et al., 2014). These results suggest that GCX is universally required for cancer survival and growth regardless of the cancer type. This was also the case in our analysis of GCX in human colon cancer in a previous study (Tachi et al., 2019). Further in-depth studies of bulky GCX, which is essential for cancer, will be necessary in the future.

In the past, several studies investigating the differences in surface lectins between bladder cancer cells and normal bladder epithelial cells have been published. For example, some studies indicated that high-grade bladder cancer cells exhibit a lack of reactivity towards the lectins WGA and DBA, while showing reactivity towards ConA. Additionally, the expression levels of glycans recognized by lectins DBA and LCA are diminished in bladder cancer cells (Guo et al., 2014). A limitation of most of these studies was that they used formalin-embedded sections, however, it is noteworthy that glycans are unstable and lectin reactivity is reduced in formalin-embedded sections compared to frozen sections (Brandon et al., 1988). Therefore, in the present study, we evaluated the glycan expression in bladder cancers using frozen sections. In addition, the pathological classification of bladder cancer and its subtypes in the past was not necessarily the same as the current classification. Our data are based on the current pathological classification and are, therefore, valuable.

VVL, a lectin isolated from Vicia villosa seeds, recognizes the GalNAc residue linked to serine or threonine in a polypeptide Tn antigen. Other glycan structures, such as Galβ1,3GalNAc-α-Ser/Thr (T antigen) and GlcNAcα1,6-GalNAc-α-Ser/Thr, including terminal α1,4- and β1,4-linked GalNAc, were also recognized by VVL, but with a weaker affinity (Tollefsen and Kornfeld, 1983; Iskratsch et al., 2009). In this study, VVL was used to identify O-GalNAcylation, and VVL recognized glycans. The expression levels of VVL-binding glycans were monitored by VVL-histochemistry and VVL-cytofluorescent staining in the previous report (Detarya et al., 2020). Furthermore, as the expression level of VVL-binding glycans is mediated via the action of GALNT, an enzyme transfers the first GalNAc from UDP-GalNAc to the serine or threonine of a polypeptide acceptor (Detarya et al., 2020). The important roles of a specific GALNT isoenzyme in carcinogenesis and metastasis in particular cancer types have been reported (Wu et al., 2011; Ding et al., 2012; Beaman and Brooks, 2014; Lin et al., 2014; Hussain et al., 2016; Lin et al., 2017; Detarya et al., 2020). Our data suggest that GALNT3, 4, 5, 6, and 11 may be the major isoforms responsible for O-GalNAcylation and VVL-binding glycan expression in high VVL-expressing matched fresh frozen samples. In addition, this study, for the first time, showed the specific VVL staining of invasive urothelial carcinomas in fresh frozen human tissues, which can maintain the glycan structure.

The transmembrane glycoprotein, Mucin1 (MUC1), is a mucin family member protein that is expressed on normal glandular epithelial cells and ductal epithelial cells and abundantly expressed on cancer cell surfaces; it is associated with cancer progression and is referred to as tumor associated MUC1 (TA-MUC1) (Nath and Mukherjee, 2014). TA-MUC1 is a tumor-associated antigen that is derived from the MUC1 protein, which is normally expressed on the surface of epithelial cells. MUC1 has a large extracellular domain that contains many serine and threonine residues that can be modified by O-glycosylation. GalNAc transferase is a key enzyme involved in O-glycosylation, as it catalyzes the first step of adding a GalNAc to the serine or threonine residue. MUC1 with the Tn epitope as a cancer marker is an antigen that is highly expressed in a form with abnormal *O*-glycosylation on the surface of a variety of cancer cells, but is not expressed or is expressed at low levels in normal tissues. Therefore, MUC1-Tn antigen can be used as an ideal target for solid tumor therapy (Gill et al., 2013). VVL recognizes GalNAc of this Tn antigen, therefore, a positive VVL staining implies the expression of Tn antigen in cancer cells. The therapeutic potential of targeting Tn-MUC1 has been studied, including the use of chimeric antigen receptor (CAR) therapies, vaccines, and antibody-drug conjugates (ADCs). Several studies have reported positive results for these approaches (Posey et al., 2016; Scheid et al., 2016; Bose and Mukherjee, 2020). The targeted Tn-MUC1 therapy may provide higher recurrence prevention and more treatment options for higher-grade bladder cancer.

A limitation of this study was the small number of cases of advanced bladder cancer, such as stage T2 or higher, lymph node metastasis, and distant metastasis, thereby limiting the generalizability of our findings. More cases are therefore needed for further analysis. Although we used the comprehensive lectin screening kits, 20 lectins, to evaluate the bulky GCX on the cancer surface in tissue specimens morphologically and microscopically, it is insufficient to clarify glycan profiles fully, An actual glycan analysis is necessary to identify the glycan structures and establish correlations with cancer progression and invasiveness in detail. Regarding techniques and methods, since GCX is fragile and is composed of diverse components, such as glycans and glycoproteins, it is unclear whether the increase in GCX assessed by SEM is similar to the increase in VVL. Further development of methods to evaluate the increase in GCX and fix its morphology is required.

## Conclusion

We were able to visualize bulky GCX in bladder cancer cells in three dimensions using SEM and analyzed glycan expression in frozen sections. The study found that a specific lectin, VVL, was present in cases of invasive urothelial carcinoma and its variant components. This lectin, which was specifically found in cases of cancer progression, has the potential to serve as a promising target for drug delivery in future clinical studies. Further experiments are required to completely understand the role of GCX in invasion and metastasis of bladder cancer.

## Data Availability

The raw data supporting the conclusion of this article will be made available by the authors, without undue reservation.
